# Instructions for the Preservation of the Health of the French Army in the East

**Published:** 1855

**Authors:** 


### INSTRUCTIONS FOR THE PRESERVATION OF THE HEALTH OF THE FRENCH ARMY IN THE EAST.

AT the commencement of the present war, the Minister of War in France consulted the Council of Health on the best sanitary measures to be adopted by the French army about to proceed to the East. That Council thereupon drew up a series of instructions, which are published in the "*Becueil de Mémoires de Médecine, de Chirurgie, et de Pharmacie Militaires*," 2ieme série, 13ieme volume. After a few introductory remarks, a topographical and meteorological account is given of the various places likely to be visited,—namely, the Archipelago, the Dardanelles, Constantinople, Gallipoli, the Bosphorus, Roumelia, the Balkan, Bulgaria, Moldavia, and Wallachia. In a second section is first given a general view of the epidemic or endemic diseases of the region, and of the times at which they prevail; and next, the diseases of each locality are more particularly specified. The third section contains hygienic and therapeutic instructions on the subjects of vaccination, dwellings (including beds, ventilation, &c.), clothing, food, condiments, drink, cleanliness, general mode of life, and the treatment of disease or wounds.

To this kind of foresight on the part of the French government is mainly due the exemption from disease with which their troops in the Crimea have been favoured. "Why were the same precautions not taken by the English government?" is the great question of the past. "Have any similar precautions even now been taken?" is the great question of the present; the answer to which is, we fear, implied in the monosyllable—*None.*

